# Intracellular Fas ligand in normal and malignant breast epithelium does not induce apoptosis in Fas-sensitive cells

**DOI:** 10.1054/bjoc.2000.1506

**Published:** 2000-12

**Authors:** G B Ragnarsson, E K Mikaelsdottir, H Vidarsson, J G Jónasson, K Ólafsdóttir, K Kristjánsdóttir, J Kjartansson, H M Ögmundsdóttir, T Rafnar

**Affiliations:** Molecular and Cell Biology Research Laboratory, Icelandic Cancer Society, P.O. Box 5420, Reykjavík, IS-125; Department of Pathology, University of Iceland, Reykjavík; Faculty of Medicine, University of Iceland; St. Josef's Hospital, Hafnarfjördur, Iceland

**Keywords:** Fas, apoptosis, breast neoplasm, tumour infiltrating lymphocytes, CD95, FasL

## Abstract

Fas ligand (FasL) is expressed on some cancers and may play a role in the immune evasion of the tumour. We used immuno-histochemistry to study the expression of Fas and FasL in tissue samples from breast cancer patients, as well as normal breast tissue. Our results show that Fas and FasL are co-expressed both in normal tissue and in breast tumours. Fas and FasL mRNA were expressed in fresh normal and malignant breast tissue, as well as cultured breast epithelium and breast cancer cell lines. Flow cytometry analysis of live cells failed to detect FasL on the surface of normal or malignant breast cells; however, both stained positive for FasL after permeabilization. Fas was detected on the surface of normal breast cells and T47D and MCF-10A cell lines but only intracellularly in other breast cell lines tested. Neither normal breast epithelium nor breast cell lines induced Fas-dependent apoptosis in Jurkat cells. Finally, 20 tumour samples were stained for apoptosis. Few apoptotic cells were detected and there was no increase in apoptotic cells on the borders between tumour cells and lymphocytes. We conclude that FasL is expressed intracellularly in both normal and malignant breast epithelium and unlikely to be important for the immune evasion of breast tumours. © 2000 Cancer Research Campaign http://www.bjcancer.com


*British Journal of Cancer* (2000) **83**(12), 1715–1721
© 2000 Cancer Research Campaign
doi: 10.1054/ bjoc.2000.1506, available online at http://www.idealibrary.com on IDEAL®

### Intracellular Fas ligand in normal and malignant breast epithelium does not induce apoptosis in Fas-sensitive cells

**GB Ragnarsson[1], EK Mikaelsdottir[1], H Vidarsson[1], JG Jónasson[2,3], K Ólafsdóttir[2], K Kristjánsdóttir[3], J Kjartansson[4], HM Ögmundsdóttir[1,3] and T Rafnar[1,3]**

[1]Molecular and Cell Biology Research Laboratory, Icelandic Cancer Society, P.O. Box 5420, IS-125, Reykjavík; [2]Department of Pathology, University of Iceland, Reykjavík, [3]Faculty of Medicine, University of Iceland; [4]St. Josef's Hospital, Hafnarfjördur, Iceland

**Summary** Fas ligand (FasL) is expressed on some cancers and may play a role in the immune evasion of the tumour. We used immunohistochemistry to study the expression of Fas and FasL in tissue samples from breast cancer patients, as well as normal breast tissue. Our results show that Fas and FasL are co-expressed both in normal tissue and in breast tumours. Fas and FasL mRNA were expressed in fresh normal and malignant breast tissue, as well as cultured breast epithelium and breast cancer cell lines. Flow cytometry analysis of live cells failed to detect FasL on the surface of normal or malignant breast cells; however, both stained positive for FasL after permeabilization. Fas was detected on the surface of normal breast cells and T47D and MCF-10A cell lines but only intracellularly in other breast cell lines tested. Neither normal breast epithelium nor breast cell lines induced Fas-dependent apoptosis in Jurkat cells. Finally, 20 tumour samples were stained for apoptosis. Few apoptotic cells were detected and there was no increase in apoptotic cells on the borders between tumour cells and lymphocytes. We conclude that FasL is expressed intracellularly in both normal and malignant breast epithelium and unlikely to be important for the immune evasion of breast tumours. © 2000 Cancer Research Campaign http://www.bjcancer.com

**Keywords:** Fas; apoptosis; breast neoplasm; tumour infiltrating lymphocytes; CD95; FasL

The Fas receptor belongs to the tumour necrosis factor (TNF) family of receptors whose activation signals the cell to undergo apoptosis (Nagata, 1997). Whereas Fas is ubiquitously expressed in the body, Fas ligand (FasL) is expressed on activated T cells and NK cells and serves an important function in the destruction of virus-infected and transformed cells. Defects in the Fas/FasL system lead to abnormal T cell development and lymphoproliferative disorders, demonstrating that apoptosis by Fas/FasL interaction is necessary for negative selection of T cells in the thymus and for keeping homeostasis of the peripheral immune system (Nagata, 1997).

Although first thought to be mostly expressed in activated T and NK cells, FasL has been found in several other cell types, i.e. epithelial cells and some tissues that enjoy a certain protection from the cellular arm of the immune system. These 'immune privileged' sites include the eye and testis, organs that could suffer irreversible damage if invaded by inflammatory cells (Bellgrau et al, 1995; Griffith et al, 1995). Early reports, showing down-regulated Fas expression and sensitivity in some human tumours caused speculations that this could be a way for tumours to evade killing by the immune system system (Strand et al, 1996). Further supporting a potential role for the involvement of Fas and FasL in tumour defence mechanisms, it was shown that some tumour cells express FasL and are able to induce apoptosis in Fas-sensitive T-cell lines (Strand et al, 1996; Niehans et al, 1997). Since these first studies were published, it has become clear that FasL is expressed in normal epithelial cells and its role in the defence of tumour cells against the immune system is far from being established (Xerri et al, 1997).

Breast tumours often contain large lymphocytic infiltrates that seem to be unable to affect the growth of the surrounding malignant cells (O'Sullivan and Lewis, 1994). This study was undertaken to analyse the expression of Fas and FasL in normal and malignant breast tissue and correlate this expression with potential functional consequences, in vivo and in vitro.

*Received 3 December 1999*
*Revised 7 August 2000*
*Accepted 14 August 2000*

*Correspondence to:* T Rafnar

### MATERIALS AND METHODS

### Patients and samples

Random samples of normal and malignant breast tissue (formalin fixed ($n = 74$) and fresh ($n = 8$)) from 74 patients, diagnosed with breast cancer in 1991–1992, as well as 5 normal samples from reduction mammoplastic operations (fresh ($n = 5$), fixed ($n = 2$)), were obtained from the Department of Pathology, University of Iceland.

### Normal breast epithelium and cell lines

Tissue samples were digested overnight with 450 IU $ml^{-1}$ collagenase (Type 1A, C9891, Sigma, St Louis, MO), at 37°C under gentle rotation. The cells were seeded into culture flasks, precoated with type I collagen (Vitrogen 100, Celtrix labs, Palo Alto, CA) and cultured in highly supplemented serum-free medium, CDM4 (chemically defined medium 4). This medium, which is used to promote growth of epithelial cells and suppress overgrowth by fibroblasts, is based on Ham's F12/Dulbecco's modified Eagle's medium (Gibco, Rockville, MD) and contains the following additives: 100 ng $ml^{-1}$ fibronectin, 2.6 ng $ml^{-1}$ sodium selenite, 20 ng $ml^{-1}$ EGF, 0.1 nM oestradiol, 0.5 μg $ml^{-1}$ hydrocortisone, 1 nM triiodothyronine, 25 μg $ml^{-1}$ transferrin, 10 μM dibutyryl cyclic AMP, 0.1 mM phosphorylethanolamine, 20 μg $ml^{-1}$ fetuin, 10 μg $ml^{-1}$ ascorbic acid, 0.01% BSA, 3.0 μg $ml^{-1}$ insulin and 10 ng $ml^{-1}$ cholera toxin (all from Sigma); 75 U $ml^{-1}$ penicillin and 75 μg $ml^{-1}$ streptomycin (Gibco). During collagenase digestion, 5% fetal bovine serum was included.

Breast cancer cell lines T47-D, MDA-MB-231, MCF-7, ZR-75-1, normal breast cell line MCF-10A and the Jurkat T cell line were obtained from American Type Culture Collection (ATCC, Manassas, VA) and grown in the media recommended by ATCC. As a positive control for surface expression of FasL, the cell lines KFL9, a human FasL-tranfectant of human myeloid leukaemia K562 (kindly provided by Dr D Kaplan, Cleveland, OH) (Smith et al, 1998) and the Jurkat variant J77 (kindly provided by Tim Hemesath, DeCode Genetics) were used. J77 was stimulated by incubation with OKT3 (ATCC, 5 μg $ml^{-1}$) for 30 minutes on ice, followed by crosslinking with goat anti-mouse Ig (Sigma) at 37˚C for 4 hours. The FasL-transfected and mock-transfected Neuro-2a cell lines were a gift from Dr A Fontana (Zurich, Switzerland) (Rensing-Ehl et al, 1995). Breast milk was obtained from healthy volunteers at the department of Obstetrics and Gynecology, the National Hospital.

### Immunohistochemistry

Immunostaining was performed on 4 μm thick tissue sections, mounted on superfrosted slides (Menzel, Germany). The formalin-fixed sections were dewaxed in xylol and rehydrated in graded ethanol solutions. Tissue sections were then incubated in citrate buffer (pH 6.0) for 2 × 5 min in an 850 W microwave oven. The sections were stained with monoclonal mouse IgG1 to Fas and FasL (Transduction Labs, Lexington, KY) and polyclonal rabbit IgG to FasL (clone Q-20; Santa Cruz Biotech, Santa Cruz, CA). The monoclonal antibodies detect the extracellular region of both proteins but the polyclonal Ab detects the intracellular domain of FasL. As a negative control, isotype matched nonspecific mouse IgG1 (PharMingen, San Diego, CA) replaced the monoclonal Ab. In the case of polyclonal rabbit Ab two controls were used: the primary Ab was preincubated with control peptide or replaced by normal rabbit serum. The antibodies were used at 2.5 μg $ml^{-1}$ (mAb) or 1 μg $ml^{-1}$ (polyclonal Ab) and incubated overnight at RT. Binding of antibodies was detected with secondary biotinylated Ab and streptavidin complexed with peroxidase (StreptABComplex DUET, DAKO, Glostrup, DK) according to the manufacturer's protocol. Double staining with monoclonal mouse IgG1 (1/100) to Fas (kindly provided by Dr Peter Krammer, German Cancer Res, Heidelberg, Germany) and the rabbit Ab to FasL, was performed on frozen tissue sections, fixed in acetone for 10 minutes. FITC labelled swine anti-rabbit (DAKO) and Cy5-linked goat anti-mouse (Amersham Pharmacia Biotech Inc, Piscataway, NJ) secondary Ab was used to detect the staining. Fas expression was detectable in tumour-infiltrating lymphocytes (TILs) in 90% of tumour samples where as FasL was expressed in TILs in 20% of tumours. 20 samples showing either strong or no expression of Fas or FasL in lymphocytes were then stained for apoptosis. Apoptosis was evaluated by TUNEL based assay (ApopTag Plus *In Situ* Detection Kit, Oncor Inc, Gaithersburg, MD) according to the manufacturer's protocol. Rat mammary tissue in involution was used as a positive control (provided with the kit). The staining was evaluated by three independent investigators as either positive or negative.

### Isolation of RNA and RT-PCR analysis

RNA was isolated from tissue samples and cell lines using the TRIzol-Reagent (Life Technologies, Inc, Rockville, MD) as described by the manufacturer. First-Strand cDNA Synthesis Kit (Amersham) was used for reverse transcription of ca 3 μg RNA. Fas-specific oligonucleotides 5′-GAA ATG AAA TCC AAA GCT TGG 3′ (forward) and 5′ - TAA TTT AGA GGC AAA GTG GCC-3′ (reverse) were used for Fas cDNA amplification which created 469-bp amplimer. The reaction mixture contained 1 μl template, 0.9 mM $MgCl_2$, 7.5 μmol of dNTP, 10 pmol of each primer and 0.5 U of Taq polymerase (Finnzyme, Espoo, Finland) in a volume of 25 μl. The thermocycler was programmed as follows: 95˚C for 2 min; 40 cycles of 95˚C for 1 min, 55˚C for 1 min, 72˚C for 1 min; followed by 72˚C for 5 min. FasL cDNA was amplified by specific primers 5′-GGA TTG GGC CTG GGG ATG TTT CA-3′ (forward) and 5′-TTG TGG CTC AGG GGC AGG TTG TTG-3′ (reverse), resulting in a 344-bp PCR product. 1–2 μl of cDNA template were added to 23–24-μl reaction mixture containing 1.5 mM $MgCl_2$, 7.5 μmol of dNTP, 7 pmol of each primer and 1.25 U of Taq polymerase. Amplification proceeded as follows: 95˚C for 2 min; 40 cycles of 94˚C for 45 s, 65˚C for 45 s, 72˚C for 1 min; 72˚C for 5 min. CD3-specific oligonucleotides 5′-GGC TGT CCT CAT CCT GGC TAT CAT-3′ (forward) and 5′-ACT GGT TTC CTT GAA GGT GGC TGT-3′ (reverse) were used to amplify CD3 cDNA, creating a 515-bp PCR product. 25 μl mixture of 1 μl template, 3 mM $MgCl_2$, 10 μmol of dNTP and 15 pmol of each primer, was heated for 5 min at 95˚C and cooled down to 80˚C. 0.5 U of Taq polymerase was added to the mixture and PCR programme proceeded as follows: 40 cycles of 95˚C for 1 min, 65˚C for 1 min, 72˚C for 2 min; followed by 72˚C for 5 min. Pyruvate dehydrogenase (PDH, positive control) was amplified by PDH-specific primers 5′-GGT ATG GAT GAG GAG CTG GA-3′ (forward) and 5′-CTT CCA CAG CCC TCG ACT AA-3′ (reverse); resulting in a 102 bp amplimer. The reaction mixture contained 1 μl template, 1.5 mM $MgCl_2$, 7.5 μmol of dNTP, 7 pmol of each primer and 0.5 U of Taq polymerase in 25-ml volume. PCR conditions were as follows: 95˚C for 2 min; 35 cycles of 95˚C for 45 s, 59˚C for 40 s, 72˚C for 1 min; followed by 5 min at 72˚C. PCR products were separated by electrophoresis on a 7.5% polyacrylamide gel, stained with ethidium bromide and visualized by UV.

### Western blotting

Normal breast cells in culture or cell lines were lysed on ice in lysis buffer (10 mM Tris-HCL, pH 7.4, 150 mMNaCl, 5 mM EDTA, 10% glycerol, 1% Triton-X100, 10 μg $ml^{-1}$ aprotinin, 1 mM PMFS, 1 mM Na-Orthovanadate, 1 mM leupeptin). Insoluble material was pelleted for 5 minutes at 14 000 ***g*** and the cell lysate was mixed with an equal volume of 2 × SDS sample buffer (4% SDS, 100 mM Tris, pH 6.8, 200 mM DTT, 0.2% bromophenol blue, 20% glycerol) and loaded onto 10% acryl-amide gels at the equivalent of $1 \times 10^6$ cells $lane^{-1}$. After electro-phoresis the proteins were transferred to Immun-Blot PVDF membrane (BioRad, Hercules, California). The blots were blocked

 © 2000 Cancer Research Campaign in 10% skim milk powder in PBS containing 0.1% Tween 20. The blots were tested with two primary FasL Abs (Transduction Labs. clone 33, PharMingen clone G247-4), followed by a horse-radish peroxidase-conjugated secondary Ab (Amersham) Immunoreactive bands were visualized by enhanced chemiluminescence (Amersham).

### FACS analysis

Before staining for surface bound Fas and FasL, cells were grown for 24 hours in the presence of 10 μM matrix metalloprotease inhibitor (MMPI KB8301, PharMingen) to prevent shedding of soluble FasL. Adherent cells were detached with 2.5 mM EDTA in PBS, washed twice in RPMI containing MMPI and dissolved in the same at a concentration of $2 \times 10^6$ cells $ml^{-1}$. 50 μl of cell suspension were stained with FITC-conjugated Fas antibody (clone DX2, DAKO) or biotinylated FasL antibody (clone NOK-1, PharMingen), followed by FITC-conjugated Streptavidin (Becton Dickinson, San Jose, CA). For detection of intracellular Fas and FasL, $1 \times 10^6$ cells were fixed and permeabilized using the Cytofix/Cytoperm kit (PharMingen), and stained with antibodies against Fas (clone 13, Transduction Labs.) or FasL (Transduction Labs, clone 33, PharMingen, clone G247-4), followed by FITC-conjugated anti-mouse IgG Ab (Becton Dickinson). Non-specific, isotype-matched primary Abs (PharMingen) were used as negative control. Apoptosis of Jurkat cells was detected using recombinant human Annexin V (CALTAG Laboratories, Burlingame, CA) as directed by the manufacturer. Analysis was performed on a FACScan flow cytometer (Becton Dickinson).

### Co-culture of normal and malignant breast epithelium and Jurkat cells

Breast cancer cell lines, normal breast epithelial cells and transfected Neuro-2a cells were seeded in 24-well tissue culture plates and grown until confluent at which time the wells contained about $8 \times 10^5$ effector cells. MMPI (10 μM) was kept in the culture for the last 24 hours before use. One half of the wells were fixed with 2% paraformaldehyde, followed by extensive washing with PBS. $2.5 \times 10^4$ Jurkat cells were added to each well in a volume of 0.5 ml and the cells were co-cultured for 24 hours. To some wells, blocking Fas Ab (clone ZB4, 250 ng $ml^{-1}$, MBL, Watertown, MA) and/or activating Fas Ab (clone CH11, 50 ng $ml^{-1}$, Oncor) were added. The Jurkat cells were analysed with respect to apoptotic and dead cells, using recombinant Annexin V and PI. All wells were set up in triplicates. After testing apoptosis at various time-points, cocultures with breast cells were harvested after 24 hours and cocultures on the FasL-transfected cell line after 4 hours.

Breast cell culture supernatants were concentrated 10 × using a filter device (Amicon) and assayed for the presence of soluble FasL (sFasL) using a sFasL-specific ELISA kit (MBL).

### RESULTS

### Fas and FasL are co-expressed in normal and malignant breast tissue

Several reports have documented downregulation of Fas and upregulation of FasL in human tumours. To examine possible changes in Fas/FasL expression in malignant breast tissue, tumour samples from 74 breast cancer patients were evaluated using immunoperoxidase staining of formalin fixed tissue (Figure 1 D, E). 71 of these samples showed positive staining for Fas and FasL, the remaining samples were inconclusive. The same pattern of staining was seen in normal breast epithelium, i.e. co-expression of Fas and FasL in luminal cells (Figure 1 A, B, G, H). Staining with monoclonal and polyclonal anti-FasL was concordant. FasL is therefore not upregulated nor is Fas downregulated in breast cancer compared to normal tissue.

To establish that the observed co-expression of Fas and FasL in normal and malignant breast tissue was not due to non-specific binding of the antibodies, RT-PCR analysis was performed on RNA isolated from normal breast epithelium in culture, fresh-frozen normal breast tissue, breast tumour samples and all breast cell lines (Figure 2). All the samples expressed both Fas and FasL mRNA (Figure 2A, B). In order to assess whether Fas and FasL detected in RNA from fresh tissue could have come from infiltrating lymphocytes, control RT-PCR was performed with primers specific for CD3 (Figure 2C). No CD3 bands were seen in any of the fresh-frozen breast tissue preparations, whereas strong amplification of CD3 mRNA was seen in Jurkat T cells and peripheral blood. It is possible that FasL could haemopoetic come from CD3 negative cells.

Finally, Western blot of total cell lysate from cultured breast epithelium from three different individuals, as well as all above mentioned breast cancer cell lines, was positive for Fas and FasL protein using antibodies from two different manufacturers (data not shown).

### FasL expression in normal and malignant breast epithelium is intracellular

FasL must be present on the cell surface to induce apoptosis in Fas-sensitive cells. The co-expression of Fas and FasL in the same cells implies that there must be a mechanism that prevents self destruction of the cells, i.e. either Fas must be insensitive to activation by FasL or FasL is not present on the cell surface. To examine the second possibility, intra- and extracellular Fas and FasL expression on various cell types was analysed by flow cytometry. Figure 3 shows representative results from these experiments. Normal cells, T47D and MCF-10A expressed Fas on the surface of the cells, whereas other cell lines did not. However, in concordance with the RNA analysis and Western blotting, Fas was detected intracellularly in all the breast cells tested. FasL was neither detected on the cell surface of normal breast epithelial cells in culture nor any of the cell lines. However, after permeabilization, all of these cell types showed clear positive staining for FasL, using two different antibodies. Thus, FasL seems to be confined to intracellular compartments under normal culture conditions.

### Fas-sensitive Jurkat cells are not killed by breast cells

It is possible that functional FasL is expressed on the surface of breast cells in such low quantity that it is not detectable by flow cytometry. To test whether FasL-expressing breast epithelial cells can induce apoptosis in Fas-sensitive Jurkat cells, these two cell types were co-cultured in the presence or absence of blocking Fas antibody (Figure 4). Neither normal epithelial cells nor malignant cell lines induced Fas-mediated apoptosis in Jurkat cells. Addition of metalloprotease inhibitor to the cultures or fixing of the breast cells with paraformaldehyde did not alter the results. Finally, to examine whether sFasL was secreted from the breast cell, culture

© 2000 Cancer Research Campaign

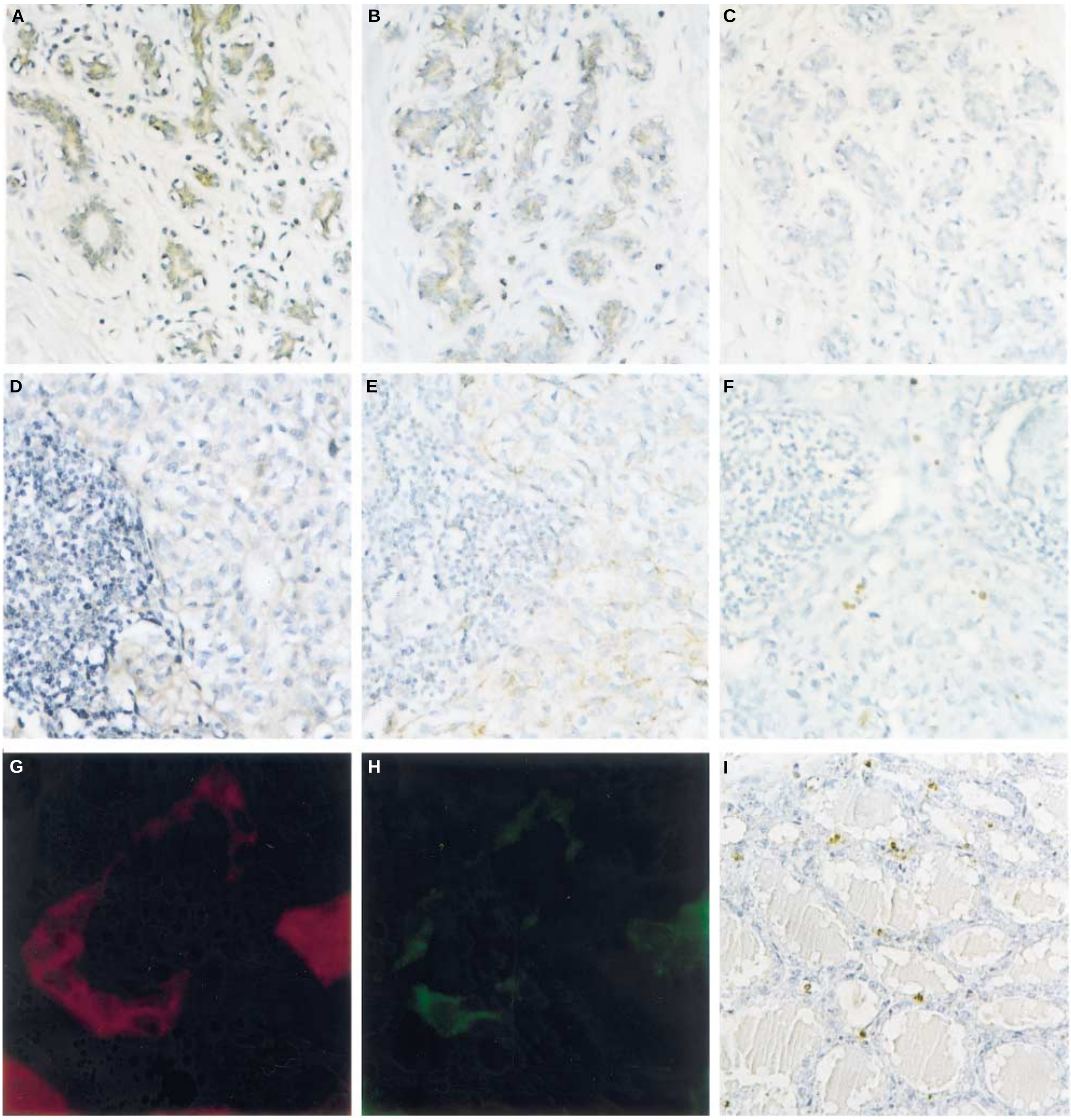

**Figure 1** Staining of Fas (**A** and **D**) and FasL (**B** and **E**) in formalin fixed normal (**A** and **B**) and malignant (**D** and **E**) breast epithelium (monoclonal Ab). Fas and FasL expression is not different in normal and tumour tissue. Co-expression on the same cells is shown by immunofluorescence costaining of Fas (**G**) and FasL (H) in cryosections of normal epithelium. Negative control (unspecific mouse IgG1) is shown in **C**. Apoptosis (**F**) is not increased in tumour infiltrating lymphocytes nor in malignant cells adjacent to the lymphocytes. Positive control (rat breast epithelium in involution) is shown in **I**. Lymphocytes: smaller cells to the left in **D–F**. (Original magnification: **A–F**, **I:** 250 ×; **G–H**: 1250×)

supernatant from normal epithelial cells and all 5 cell lines was concentrated 10 × and analysed by sFasL-specific ELISA. sFasL was not detected in any of the culture supernatants (data not shown).

### Analysis of apoptosis in breast tumours

Our results suggest that FasL expression may not be a factor in protecting breast tumour cells from the immune system, i.e. as long as FasL expression is intracellular it can exert little effect on incoming lymphocytes. As a final step in our analysis, we evaluated apoptosis in 20 paraffin-embedded breast tumour samples that had detectable lymphocytic infiltrates and had been stained previously for Fas and FasL (Figure 1F). A few apoptotic lymphocytes were detected in all samples; however, none of the samples showed marked apoptosis in areas where tumour and lymphocytes meet. Thus, in situ analysis of apoptosis in breast tumours does not indicate that tumour cells are causing apoptosis in adjacent lymphocytes or vice versa.

 *© 2000 Cancer Research Campaign*

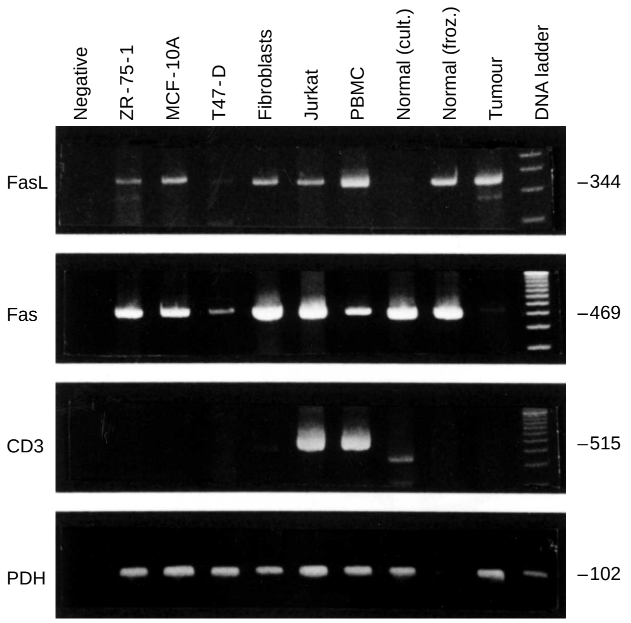

**Figure 2** RT-PCR analysis of FasL, Fas, CD3 and pyruvate dehydrogenase expression in various breast cells. Samples are ordered in the following way: 1, negative control (no cDNA); 2, ZR-75-1; 3, MCF-10A; 4, T47D; 5, cultured fibroblasts; 6, Jurkat cells; 7, peripheral blood mononuclear cells (PBMC); 8, normal breast tissue (primary culture); 9, normal breast tissue (frozen); 10, tumour breast tissue (frozen); 11, DNA ladder. Very faint band was detected for: FasL in T47D and normal cultured cells and Fas in tumour tissue

### DISCUSSION

Breast tumours often contain large infiltrates of lymphocytes but the functional or prognostic significance of these cells is not clear despite decades of study (Stewart and Tsai, 1993; O'Sullivan and Lewis, 1994). Although it has been possible to isolate T cells that have cytolytic activity against the tumour in vitro, these cells seem to be unable to control tumour growth in vivo (Whiteside et al, 1986; Jerome et al, 1991). Several reasons have been suggested for this failure of the immune system; downregulation of MHC antigens on the tumour, secretion of immunosuppressive substances by tumour cells or lack of costimulation, resulting in anergy of T lymphocytes, can all play a role in the tumour's escape from immune attack (Baskar et al, 1996; Chouaib et al, 1997; Garrido et al, 1997).

The discovery that a subset of primary tumours, as well as cell lines, may reduce their expression of Fas and start to express FasL suggested yet another way for solid tumours to defend themselves against activated lymphocytes (O'Connell et al, 1999a). FasL on tumour cell lines and tumour tissue sections, can cause apoptosis in Fas-sensitive Jurkat cells (Strand et al, 1996; Niehans et al, 1997) and one report suggested an increase in apoptosis of tumour-infiltrating lymphocytes (TIL) in FasL-expressing oesophageal carcinoma (Bennett et al, 1998). It is worth noting that some controversy remains about FasL expression in melanomas (Hahne et al, 1996; Chappell et al, 1999). We examined Fas and FasL expression in both normal and malignant breast tissue and found that all tumour samples that showed conclusive staining, co-expressed both receptors also shown by other recent study (O'Connell et al, 1999b). However, unexpectedly, we found that normal breast epithelium also expresses both receptors; therefore, FasL expression on cancerous tissue was not an adaptation to

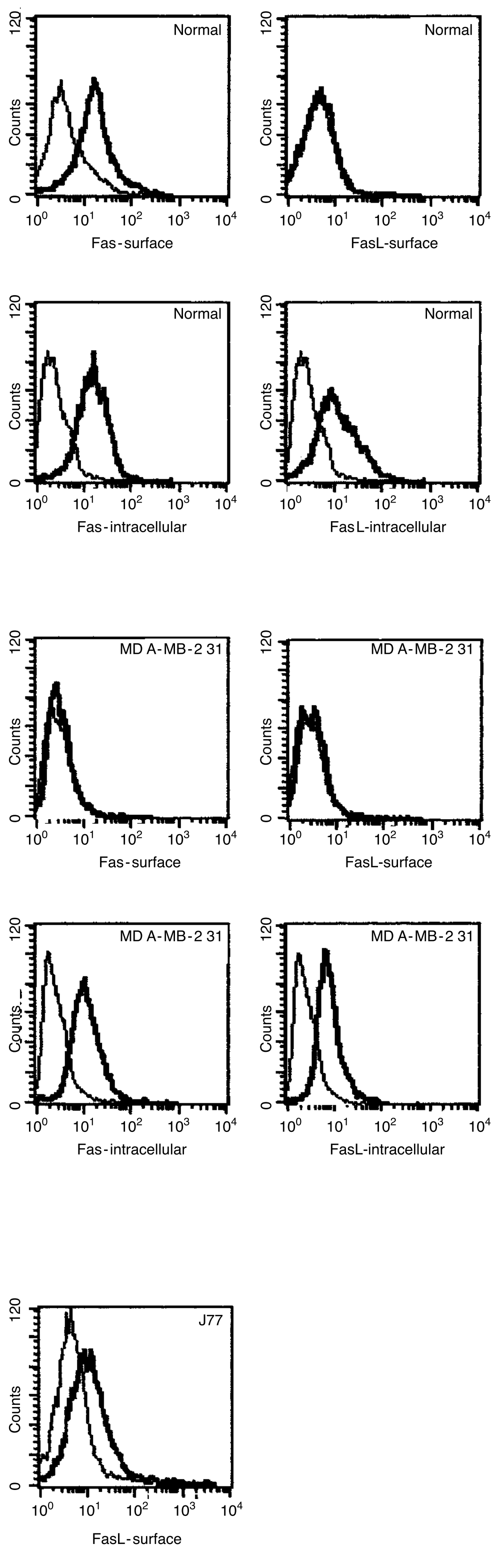

**Figure 3** Flow cytometry analysis of Fas and FasL expression in normal epithelial cells in culture and a representative cell line, MDA-MB-231. FasL was detected on the surface of the Jurkat variant J77 after CD3 crosslinking (non-shaded curve) and on the KFL9 cell line (data not shown)

evade an immune attack. Furthermore, Fas is not downregulated in breast cancer which is in accord with a previous report (Leithäuser et al, 1993). This pattern of expression was further confirmed by Western blot and RT-PCR. FACS-analysis of normal and cancer

© 2000 Cancer Research Campaign 

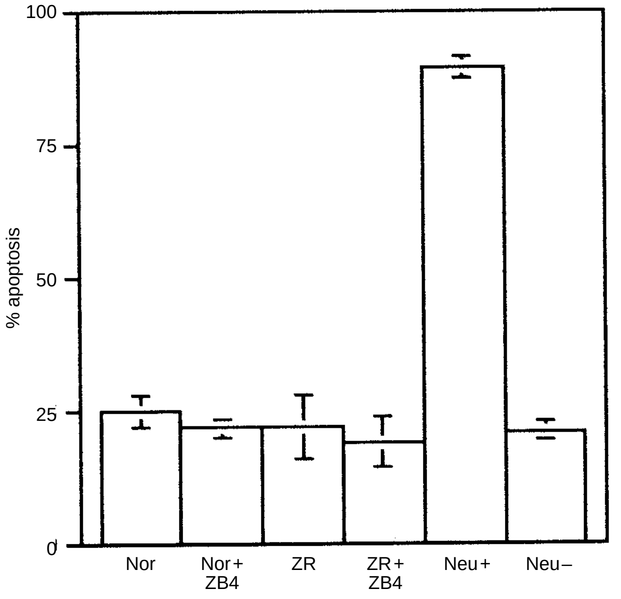

**Figure 4** Percent apoptosis of Fas-sensitive Jurkat cells after 24 hour co-culture with normal breast cells (Nor) and ZR-75-1 (ZR) in the absence or presence of blocking Fas Ab (ZB4). For controls, Jurkat cells were cocultured with FasL-transfected Neuro-2a cells (Neu+) and mock-transfected Neuro-2a cells (Neu−)

cells demonstrated that FasL is only expressed intracellularly thus unable to kill neighbouring cells. In co-culture, neither normal nor malignant breast epithelium, induced apoptosis in Fas-sensitive Jurkat cells. Similar results were published by Kontny et al who found that Ewing sarcoma cells only expressed FasL intracellularly and did not induce apoptosis in Jurkat cells (Kontny et al, 1998). If sFasL is released by the tumour cells this could prevent killing of Fas-positive T-lymphocytes (O'Connell et al, 1999a) but sFasL was not detected in culture supernatants from breast cells. When the tissue samples were stained for apoptosis, we found no indication that the tumour was causing apoptosis in infiltrating lymphocytes or vice versa. Our conclusion is, therefore, that lymphocytes and cancerous cells coexist in the tumours.

Our data raise a number of questions about the role of FasL expression in normal breast epithelium. The role of Fas/FasL in cellular homeostasis is well known in the immune system but not in epithelial tissue. Co-expression of Fas and FasL has been reported in other epithelial tissues, especially at sites of rapid turnover, e.g. in colon (Iwamoto et al, 1996; Xerri et al, 1997). The breast is a site of a rapid turnover of cells, through each menstrual cycle, lactation and involution (Russo and Russo, 1987). Thus, Fas and FasL might play a role in keeping the finely tuned balance between cellular proliferation and cell death in the breast. Interestingly, a recent report demonstrated the existence of sFasL and sFas in breast milk (Srivastava and Srivastava, 1999), suggesting that these receptors do have a functional role in the normal physiology of the breast.

We conclude that both normal and malignant breast tissue express both Fas and FasL, but functional FasL is not present on the surface of the cells and therefore has no effect on the prognosis of the breast cancer patients. Furthermore, FasL expression on breast tumour cells does not seem to cause apoptosis in neighbouring lymphocytes. Our results do not support an active role for FasL in the escape of breast tumours from immune attack.

### ACKNOWLEDGEMENTS

This work was supported by the Icelandic Cancer Society and the Icelandic Research Council.

### REFERENCES

Baskar S, Clements VK, Glimcher LH, Nabavi N and Ostrand-Rosenberg S (1996) Rejection of MHC class II-transfected tumor cells requires induction of tumor-encoded B7-1 and/or B7-2 costimulatory molecules. *J Immunol* **156**: 3821–3827

Bellgrau D, Gold D, Selawry H, Moore J, Franzusoff A and Duke RC (1995) A role for CD95 ligand in preventing graft rejection. *Nature* **377**: 630–632

Bennett MW, O'Connell J, O'Sullivan GC, Brady C, Roche D, Collins JK and Shanahan F (1998) The Fas counterattack in vivo: apoptotic depletion of tumor-infiltrating lymphocytes associated with Fas ligand expression by human esophageal carcinoma. *J Immunol* **160**: 5669–5675

Chappell DB, Zaks TZ, Rosenberg SA and Restifo NP (1999) Human melanoma cells do not express Fas (Apo-1/CD95) ligand. *Cancer Res* **59**: 59–62

Chouaib S, Asselin-Paturel C, Mami-Chouaib F, Caignard A and Blay JY (1997) The host-tumor immune conflict: from immunosuppression to resistance and destruction. *Immunol Today* **18**: 493–497

Garrido F, Ruiz-Cabello F, Cabrera T, Perez-Villar JJ, Lopez-Botet M, Duggan-Keen M and Stern PL (1997) Implications for immunosurveillance of altered HLA class I phenotypes in human tumours. *Immunol Today* **18**: 89–95

Griffith TS, Brunner T, Fletcher SM, Green DR and Ferguson TA (1995) Fas ligand-induced apoptosis as a mechanism of immune privilege. *Science* **270**: 1189–1192

Hahne M, Rimoldi D, Schröter M, Romero P, Schreier M, French LE, Schneider P, Bornard T, Fontana A, Lienard D, Cerottini JC and Tschopp J (1996) Melanoma cell expression of Fas (Apo-1/CD-95) ligand: implication for Tumor immune escape. *Science* **274**: 1363–1366

Iwamoto M, Koji T, Makiyama K, Kobayashi N and Nakane PK (1996) Apoptosis of crypt epithelial cells in ulcerative colitis. *J Pathol* **180**: 152–159

Jerome KR, Barnd DL, Bendt KM, Boyer CM, Taylor-Papadimitriou J, McKenzie IF, Bast RC Jr. and Finn OJ (1991) Cytotoxic T-lymphocytes derived from patients with breast adenocarcinoma recognize an epitope present on the protein core of a mucin molecule preferentially expressed by malignant cells. *Cancer Res* **51**: 2908–2916

Kontny HU, Lehrnbecher TM, Chanock SJ and Mackall CL (1998) Simultaneous expression of Fas and nonfunctional Fas ligand in Ewing's sarcoma. *Cancer Res* **58**: 5842–5849

Leithäuser F, Dhein J, Mechtersheimer G, Koretz K, Brüderlein S, Henne C, Schmidt A, Debatin K-M, Krammer PH and Möller P (1993) Constitutive and induced expression of APO-1, a new member of the nerve growth factor/tumor necrosis factor receptor superfamily, in normal and neoplastic cells. *Laboratory Investigation* **69**: 415–429

Nagata S (1997) Apoptosis by death factor. *Cell* **88**: 355–365

Niehans GA, Brunner T, Frizelle SP, Liston JC, Salerno CT, Knapp DJ, Green DR and Kratzke RA (1997) Human lung carcinomas express Fas ligand. *Cancer Res* **57**: 1007–1012

O'Connell J, Bennett MW, O'Sullivan GC, Collins JK and Shanahan F (1999a) The Fas counterattack: cancer as a site of immune privilege. *Immunol Today* **20**: 46–52

O'Connell J, Bennett MW, O'Sullivan GC, O'Callaghan J, Collins JK and Shanahan F (1999b) Expression of Fas (CD95/APO-1) ligand by human breast cancers: significance for tumor immune privilege. *Clin Diagn Lab Immunol* **6**: 457–463

O'Sullivan C and Lewis CE (1994) Tumour-associated leucocytes: friends or foes in breast carcinoma. *J Pathol* **172**: 229–235

Rensing-Ehl AK, Frei B, Flury B, Matiba SM, Mariani M, Weller P, Aebischer PH, Krammer and Fontana A (1995) Local Fas/APO-1 (CD95) ligand-mediated tumor cell killing in vivo. *Eur J Immunol* **25**: 2253

Russo J and Russo I (1987) Development of the human mammary gland. In: Neville M and Daniel C (eds) *The mammary gland: development, regulation and function*. Plenum Press: New York, pp 625

Smith D, Sieg S, Kaplan D (1998) Aberrant detection of cell surface Fas ligand with anti-peptide antibodies. *J Immunol* **160**: 4159

Srivastava MD and Srivastava BI (1999) Soluble Fas and soluble Fas ligand proteins in human milk: possible significance in the development of immunological tolerance. *Scand J Immunol* **49**: 51–54

 *© 2000 Cancer Research Campaign*

Stewart THM and Tsai SCJ (1993) The possible role of stromal cell stimulation in worsening the prognosis of a subset of patients with breast cancer. *Clin Exp Metastasis* **11**: 295–305

Strand S, Hofmann WJ, Hug H, Müller M, Otto G, Strand D, Mariani SM, Stremmel W, Krammer PH and Galle PR (1996) Lymphocyte apoptosis induced by CD95 (APO-1/Fas) ligand-expressing tumor cells – a mechanism of immune evasion. *Nature Medicine* **2**: 1361–1366

Whiteside TL, Miescher S, Hurlimann J, Moretta L and von Fliedner V (1986) Clonal analysis and in situ characterization of lymphocytes infiltrating human breast carcinomas. *Cancer Immunol Immunother* **23**: 169–178

Xerri L, Devilard E, Hassoun J, Mawas C and Birg F (1997) Fas ligand is not only expressed in immune privileged human organs but is also coexpressed with Fas in various epithelial tissues. *Mol Pathol* **50**: 87–91

*© 2000 Cancer Research Campaign*
